# Supplementary material for: Curvature‐Controlled Polarization in Adaptive Ferroelectric Membranes
Source: Small. 2025 Sep 18;21(41):e06338. doi: 10.1002/smll.202506338 (PMC12530018; doi:10.1002/smll.202506338)
Supplement: Supplementary file 1 — Supporting Information [file SMLL-21-e06338-s001.pdf]

NANO · MICRO
small

Supporting Information

for *Small*, DOI 10.1002/smll.202506338

Curvature-Controlled Polarization in Adaptive Ferroelectric Membranes

Greta Segantini, Ludovica Tovaglieri, Chang Jae Roh, Chih-Ying Hsu, Seongwoo Cho, Ralph Bulanadi, Petr Ondrejkovic, Pavel Marton, Jirka Hlinka, Stefano Gariglio, Duncan T.L. Alexander, Patrycja Paruch, Jean-Marc Triscone, Céline Lichtensteiger and Andrea D. Caviglia**

Supporting Information

Greta Segantini† Ludovica Tovaglieri† Chang Jae Roh† Chih-Ying Hsu Seongwoo Cho Ralph Bulanadi Petr Ondrejkovic Pavel Marton Jirka Hlinka Stefano Gariglio Duncan T.L. Alexander Patrycja Paruch Jean-Marc Triscone Céline Lichtensteiger Andrea D. Caviglia**

Dr. Greta Segantini*
Department of Quantum Matter Physics, University of Geneva, 24 Quai Ernest-Ansermet, CH-1211 Geneva 4, Switzerland.
Email Address:greta.segantini@unige.ch
Ludovica Tovaglieri
Department of Quantum Matter Physics, University of Geneva, 24 Quai Ernest-Ansermet, CH-1211 Geneva 4, Switzerland.
Dr. Chang Jae Roh
Department of Quantum Matter Physics, University of Geneva, 24 Quai Ernest-Ansermet, CH-1211 Geneva 4, Switzerland.
Dr. Chih-Ying Hsu
Department of Quantum Matter Physics, University of Geneva, 24 Quai Ernest-Ansermet, CH-1211 Geneva 4, Switzerland.
Electron Spectrometry and Microscopy Laboratory (LSME), Institute of Physics (IPHYS), Ecole Polytechnique Fédérale de Lausanne (EPFL), CH-1015 Lausanne, Switzerland.
Dr. Seongwoo Cho
Department of Quantum Matter Physics, University of Geneva, 24 Quai Ernest-Ansermet, CH-1211 Geneva 4, Switzerland.
Dr. Ralph Bulanadi
Department of Quantum Matter Physics, University of Geneva, 24 Quai Ernest-Ansermet, CH-1211 Geneva 4, Switzerland.
Dr. Petr Ondrejkovic
Institute of Physics of the Czech Academy of Sciences, Na Slovance 2, Praha 8, 182 00, Czech Republic.
Dr. Pavel Marton
Institute of Mechatronics and Computer Engineering, Technical University of Liberec, Studentska 2, Liberec, 46117, Czech Republic.
Institute of Physics of the Czech Academy of Sciences, Na Slovance 2, Praha 8, 182 00, Czech Republic.
Dr. Jirka Hlinka
Institute of Physics of the Czech Academy of Sciences, Na Slovance 2, Praha 8, 182 00, Czech Republic.
Dr. Stefano Gariglio
Department of Quantum Matter Physics, University of Geneva, 24 Quai Ernest-Ansermet, CH-1211 Geneva 4, Switzerland.
Dr. Duncan T.L. Alexander
Electron Spectrometry and Microscopy Laboratory (LSME), Institute of Physics (IPHYS), Ecole Polytechnique Fédérale de Lausanne (EPFL), CH-1015 Lausanne, Switzerland.
Prof. Patrycja Paruch
Department of Quantum Matter Physics, University of Geneva, 24 Quai Ernest-Ansermet, CH-1211 Geneva 4, Switzerland.
Prof. Jean-Marc Triscone
Department of Quantum Matter Physics, University of Geneva, 24 Quai Ernest-Ansermet, CH-1211 Geneva 4, Switzerland.
Dr. Céline Lichtensteiger
Department of Quantum Matter Physics, University of Geneva, 24 Quai Ernest-Ansermet, CH-1211 Geneva 4, Switzerland.
Prof. Andrea D. Caviglia*
Department of Quantum Matter Physics, University of Geneva, 24 Quai Ernest-Ansermet, CH-1211 Geneva 4, Switzerland.

1 Symmetry Analysis and Characterisation by SHG

The observed SHG patterns were analyzed by considering the SHG responses given by $I_{ED}^{2\omega} = |P_{ED}^{2\omega}|^2 = |\chi_{ijk} E_j E_k|^2$ [1]. Here, E_i denotes the fundamental wave's electric field with a polarization state along i-axis and χ_{ijk} represents the second-order nonlinear susceptibility tensor. To characterize the crystal symmetry of $PbTiO_3$, we took into account the two-electric dipole contributions of the polar tetragonal structures with point group *4mm* and *m4m* (see SI Figure S6a and S6b), having broken inversion symmetry along out-of-plane axis and in-plane axis, respectively [2]. Among the two point groups, the in-plane polar tetragonal one with a point group *m4m* is valid, while another case with a point group *4mm* is forbidden. The reason for this is that the out-of-plane components of the light polarization states are limited in normal incidence geometry. Therefore, the observed SHG responses reflect the inversion symmetry breaking along the in-plane direction, monitoring only the ferroelectric a domains, but not the c ones.

Although we focused the fundamental wave to a diffraction limit, i.e. sub-micrometer scale, the probing size is still larger than the typical ferroelectric domain size with a width of a few tens-hundreds of nanometers. Accordingly, the monitored SHG responses reflect ferroelectric multi-domain states. SI Figure S6c shows the possible eight combinations of ferroelectric domain states in $PbTiO_3$, such as a/c and a_1/a_2 domain configurations. For the a/c domain structure, only the a domain could be detected due to the normal-incidence optical geometry. Hence, SHG response technically detects only a single in-plane ferroelectric domain response within the a/c domain structure.

For the simulation and the fitting process, we considered a coherently contributed SHG response by two electric dipole sources as $I_{ED}^{2\omega} = |P_{ED1}^{2\omega} + P_{ED2}^{2\omega}|^2$, where $P_{ED1}^{2\omega}$ and $P_{ED2}^{2\omega}$ denote the SHG responses coming from the single ferroelectric states, consisting in the superdomain structure.[3, 4] SI Figure S6d shows the simulated SHG patterns in perpendicular polarization states of the fundamental and SHG waves, respectively. For the simulation, we set the second-order nonlinear susceptibility tensors set to $\chi_{xxx} = 0$, $\chi_{xyy} = 1$, $\chi_{yyx} = 2$, and $\chi_{yxy} = 0$ for in-plane a_1-domain state and $\chi_{yyy} = 0$, $\chi_{yxx} = 1$, $\chi_{xxy} = 2$, and $\chi_{xyx} = 0$ for in-plane a_2-domain state, respectively.

2 Determination of Ripple Parameters

Ripples were classified by parameters acquired from the topography image in Figure 3a of the main manuscript. To do so, the image was first rotated such that the ripples were oriented primarily along vertical and horizontal axes. SciPy [5] was then used to identify all local maxima on each line with at least 5-nm prominence and 380-nm separation from an adjacent peak. Each peak was then fitted to a Gaussian curve, modeled as:

$$f(x) = A \exp\left[\frac{-(x-\mu)^2}{2\sigma^2}\right] + z_0 \tag{1}$$

where A is the height of the ripple above the baseline z_0, μ is the centre of the ripple, and σ is the standard deviation. The full-width at half maximum (FWHM) above the baseline could subsequently be determined as:

$$\mathrm{FWHM} = 2\sqrt{2\ln(2)}\sigma \tag{2}$$

The radius of curvature R of an arbitrary function $f(x)$ is

$$R = \left|\frac{(1+f'(x)^2)^{\frac{3}{2}}}{f''(x)}\right| \tag{3}$$

and subsequently, the radius of curvature at the crest of a Gaussian fits is $(x = \mu)$:

$$R = \frac{\sigma^2}{A} \tag{4}$$

The Gaussian fit parameters were collected for each peak, and this process was repeated on each line on both horizontal and vertical axes.

Two peaks each fitted to a Gaussian curve were considered part of the same ripple if:

1. Each fit was separated by less than 5 lines ($\sim$ 146 nm);
2. the difference in μ between both fits were less than 5 px ($\sim$ 146 nm); and
3. A did not change by more than 25%.

The height of a ripple was calculated as the mean of A of the Gaussian fits that compose the central 50% of that ripple, and the error in this value was calculated as the standard deviation of this set. The FWHM and curvature of the ripple was likewise calculated from the mean and standard deviation of the FWHM and curvature of the constituent Gaussian fits.

Ripples with a distance from end-to-end that spanned at least 20 lines ($\sim$ 586 nm) and propagated within 20° of the horizontal or vertical axes were analyzed. The height of these ripples as a function of FWHM is shown in Figure 3b and reproduced in SI Figure S8a, while the radius of curvature as a function of FWHM is shown in SI Figure S8b.

The characteristic radius of curvature of primary ripples, $R_{min} = 1.55 \pm 0.18\mu$m, is marked by the orange band in both plots. Primary ripples lie on or near this band, with low uncertainty, while secondary ripples present larger radii of curvature, lower heights, and larger uncertainties in these properties.

The parameters of some representative primary and secondary ripples are shown in SI Table S1 and SI Figure S9i. These ripples are shown in profile in SI Figure S9(a-h).

Table S1: Ripple Parameters

No.	Height (nm)	FWHM (nm)	Curvature (μm)	Type
1	56.4 ± 3.2	640 ± 20	1.29 ± 0.07	Primary
2	56.1 ± 2.8	740 ± 40	1.72 ± 0.17	Primary
3	34.5 ± 2.7	550 ± 60	1.58 ± 0.26	Primary
4	32.9 ± 3.1	530 ± 40	1.46 ± 0.14	Primary
5	17.8 ± 1.8	410 ± 60	1.87 ± 0.51	Secondary
6	13.8 ± 1.4	440 ± 80	2.52 ± 0.90	Secondary
7	6.9 ± 0.4	330 ± 50	3.00 ± 0.87	Secondary
8	4.7 ± 0.4	270 ± 30	2.98 ± 0.72	Secondary

3 Supporting Information Figures

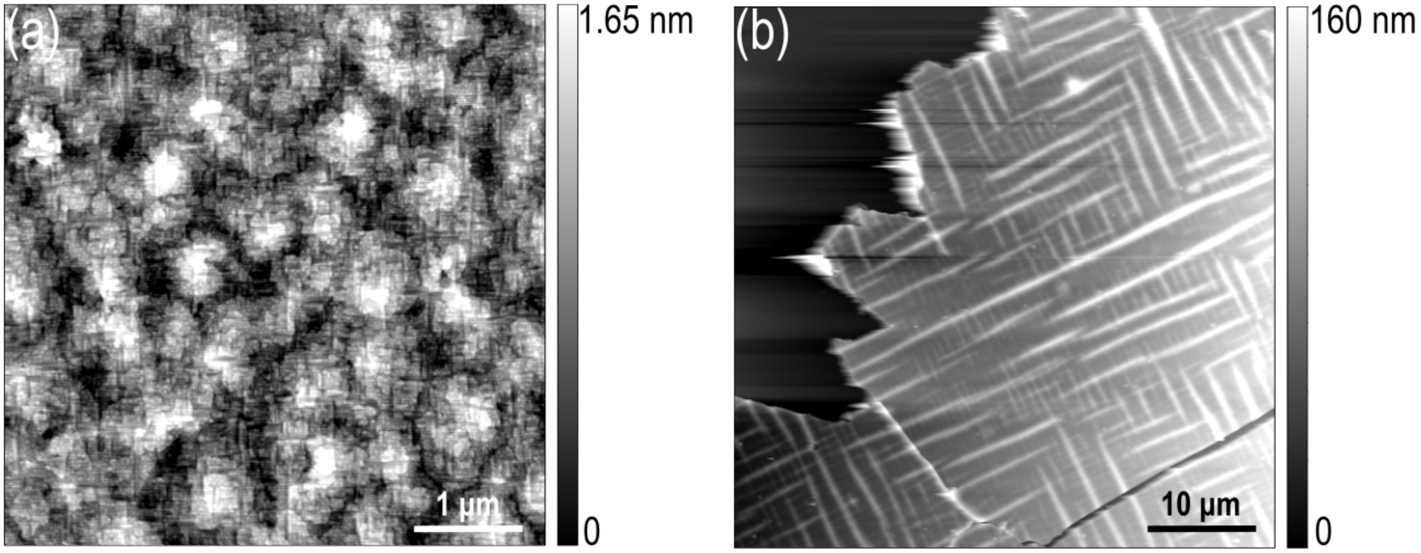

Figure S1: (a) Topography images of the heterostructure $PbTiO_3/SrRuO_3/SrTiO_3/Sr_3Al_2O_6/SrTiO_3(001)$ before lift-off and transfer. (b) Topography image of the $PbTiO_3/SrRuO_3$ flake transferred onto the $Nb:SrTiO_3(001)$ substrate.

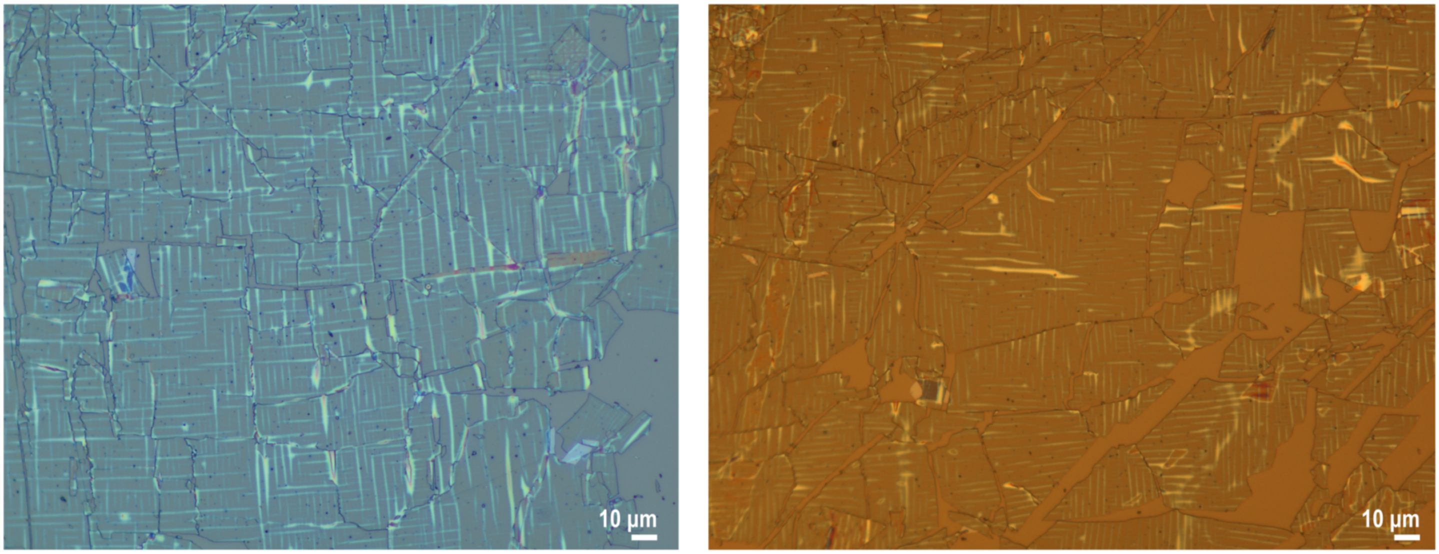

Figure S2: Optical images, acquired in different regions of the sample, of the $PbTiO_3/SrRuO_3$ membrane transferred onto the $Nb:SrTiO_3(001)$ substrate. The ripple pattern on the transferred flakes is clearly distinguishable.

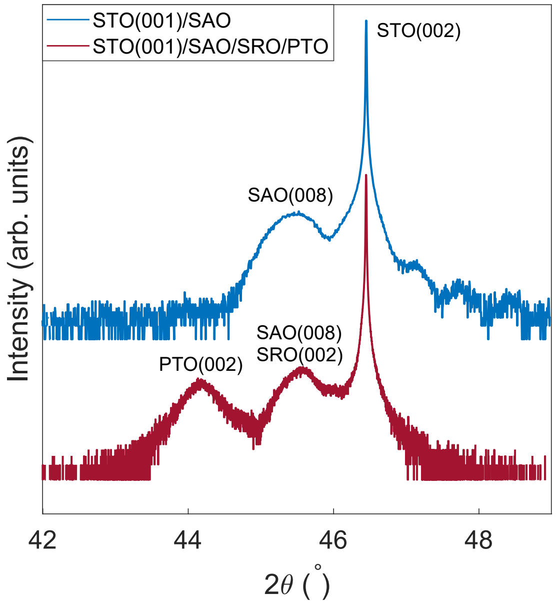

Figure S3: X-ray diffraction patterns of the heterostructures $SrTiO_3(001)/Sr_3Al_2O_6$ and $PbTiO_3/SrRuO_3/Sr_3Al_2O_6/SrTiO_3(001)$ before lift-off and transfer of the membrane on the Nb:$SrTiO_3(001)$ substrate.

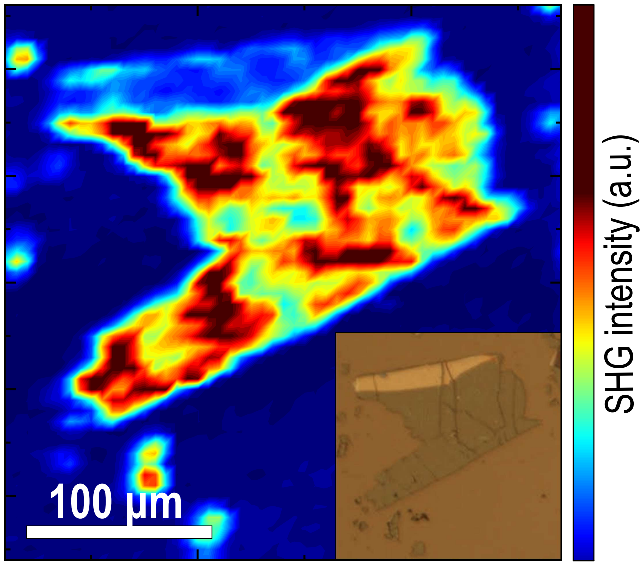

Figure S4: SHG image covering the entire $PbTiO_3/SrRuO_3$ flake area and the $SrTiO_3$ substrate. The inset shows the optical image of the corresponding region.

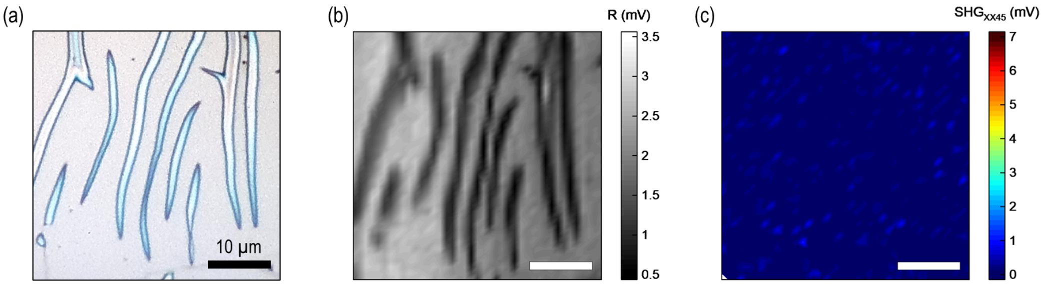

Figure S5: SHG microscopy result on $SrRuO_3$ (22 nm)/$SrTiO_3$ (1.6 nm) membrane. (a) Optical microscope image. (b) 2D reflection image. (c) 2D SHG image of the corresponding region of (b). The origin of the observed ripples is attributed to the membrane transfer process onto the target substrate, as they do not exhibit any periodic pattern or alignment with the crystallographic axes.

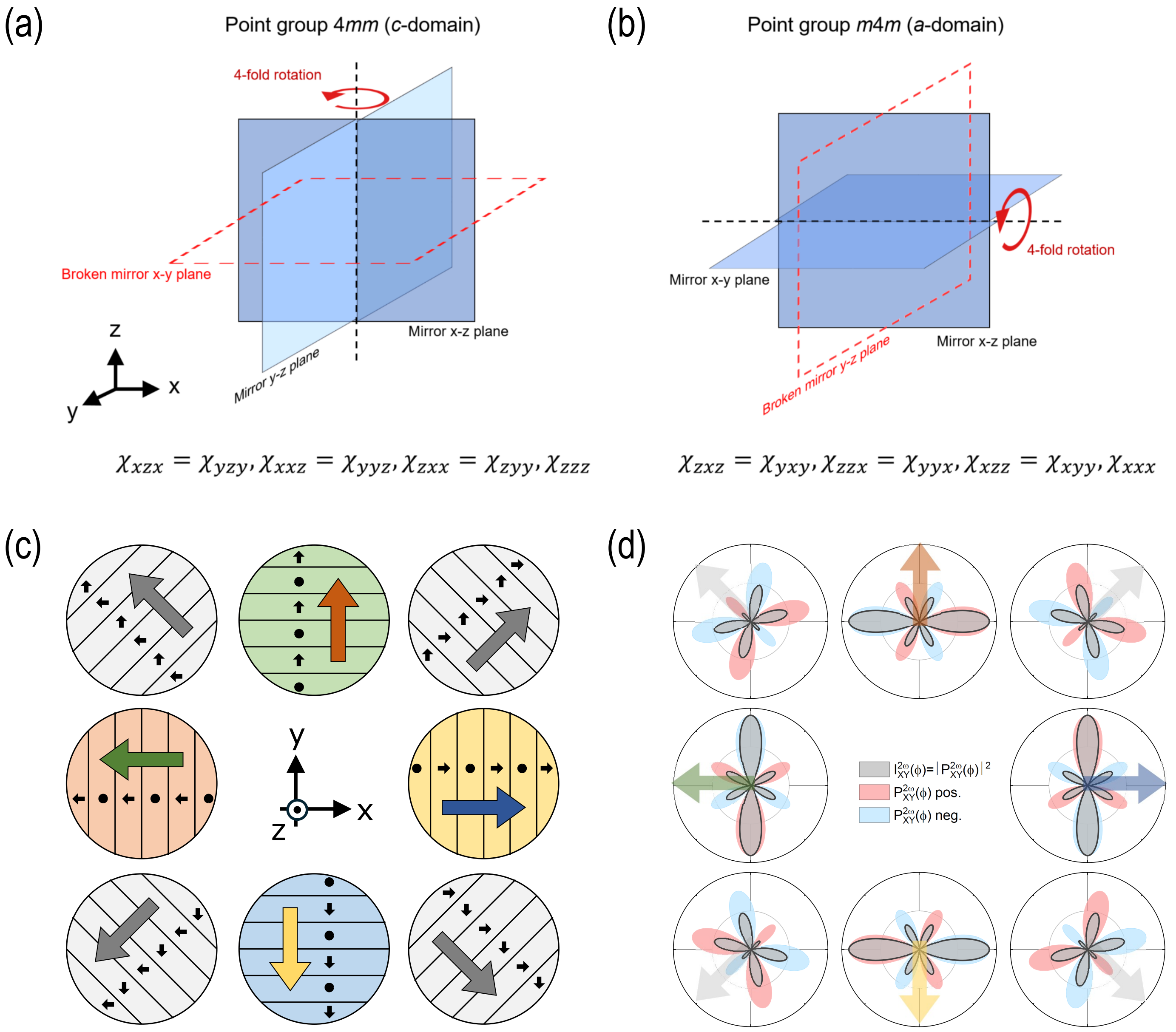

$\chi_{xzx} = \chi_{yzy}, \chi_{xxz} = \chi_{yyz}, \chi_{zxx} = \chi_{zyy}, \chi_{zzz}$

$\chi_{zxz} = \chi_{yxy}, \chi_{zzx} = \chi_{yyx}, \chi_{xzz} = \chi_{xyy}, \chi_{xxx}$

Figure S6: Symmetry characterization and SHG simulation of ferroelectric superdomain states. (a,b) Candidate point groups for polar tetragonal structures with broken inversion symmetry along (a) out-of-plane and (b) in-plane directions, respectively. (c) Possible polarization configurations consisting of a/c (colored) and a_1/a_2 (gray) domains. The black colored arrow and dot describe a single ferroelectric domain, and the large arrows indicate the in-plane net polarization for each configuration. (d) Simulated SHG polar patterns for each configuration represented in (c).

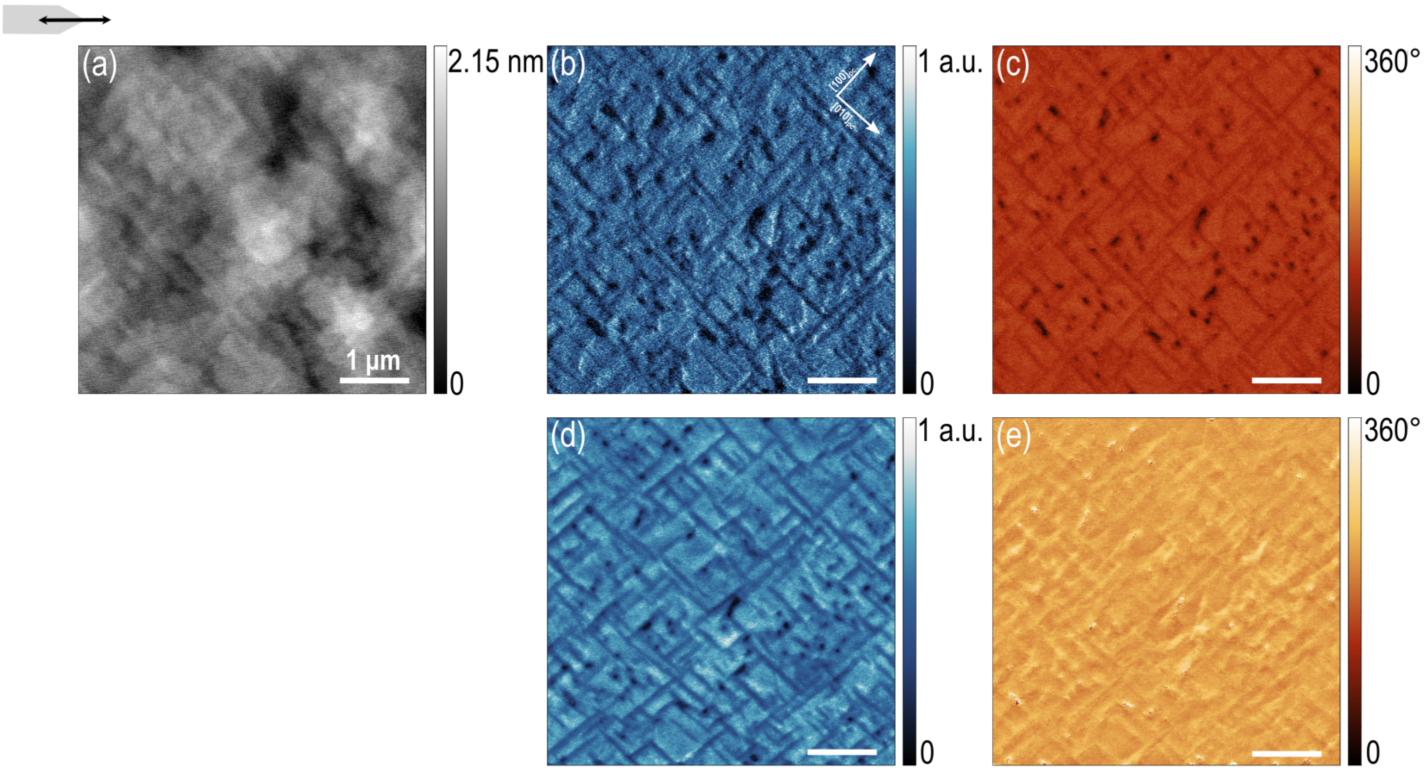

Figure S7: PFM analysis performed on the $PbTiO_3/SrRuO_3/Sr_3Al_2O_6/SrTiO_3(001)$ heterostructure before the lift-off and transfer of the membrane. (a) Topography image. (b,c) Vertical PFM amplitude and phase signals. (d,e) Lateral PFM amplitude and phase signals. These indicate the presence of an a/c domain configuration.

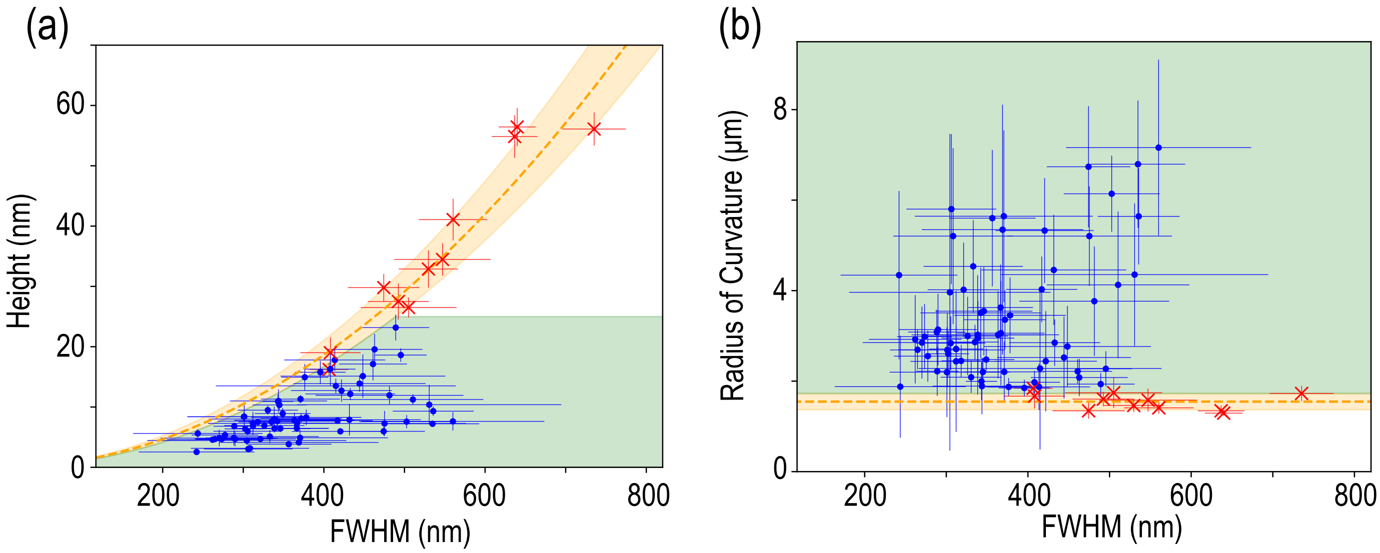

Figure S8: Parameters of ripple shown in Figure 3a. (a) The ripple height as a function of FWHM.(b) The radius of curvature of the ripple as a function of FWHM. The orange band on both plots shows a radius of curvature of 1.55 ± 0.18 μm corresponding chiefly to the primary ripples, while the green region highlights regions in where ripples present a larger radius of curvature and a height less than 25 nm.

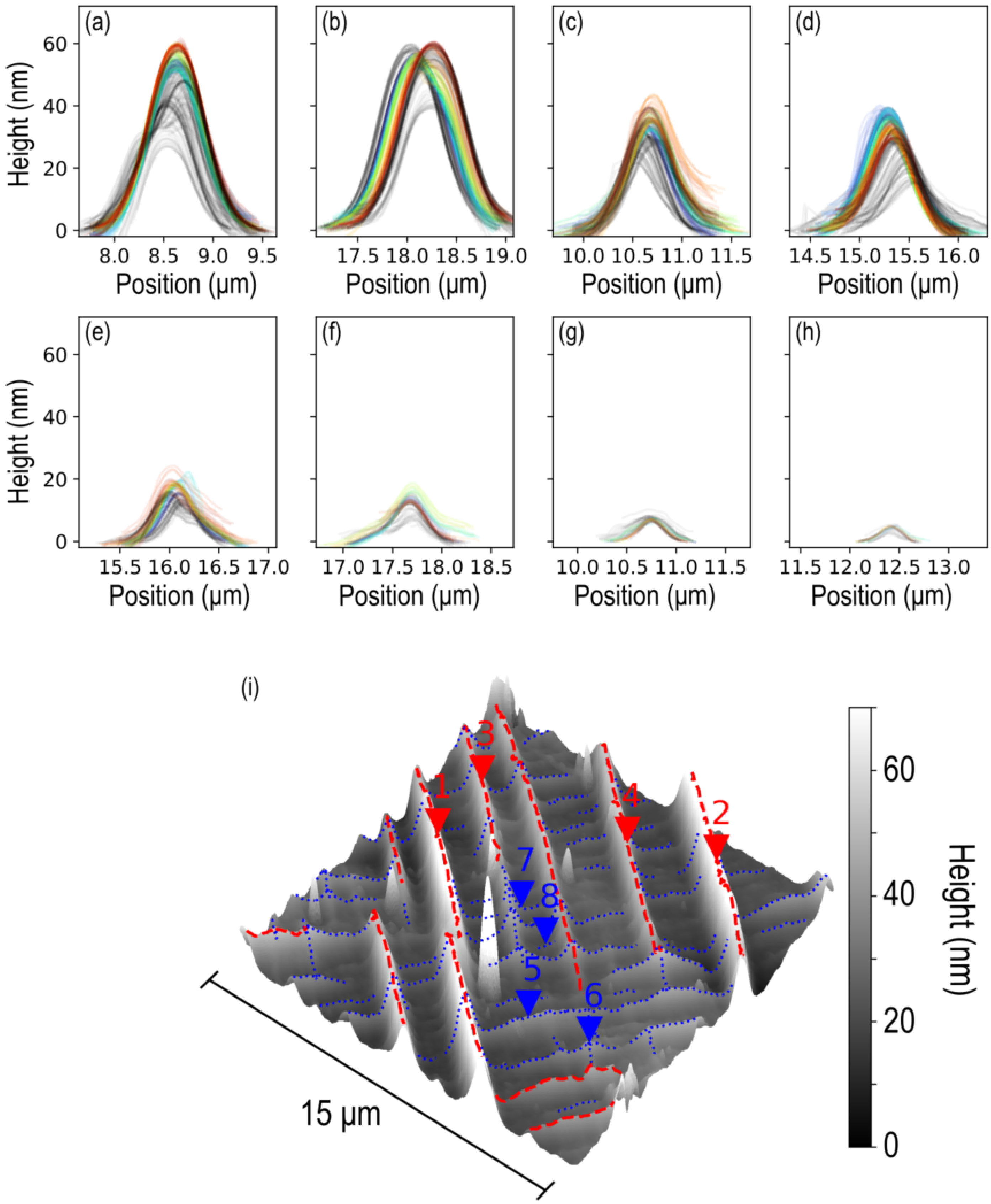

Figure S9: Waterfall plots showing line profiles of (a–d) four primary ripples, and (e–h) four secondary ripples. Coloured lines show profiles for the central 50% of the ripple, while black lines show line profiles for regions outside of this range. (i) shows the position of these ripples on the original topography map.

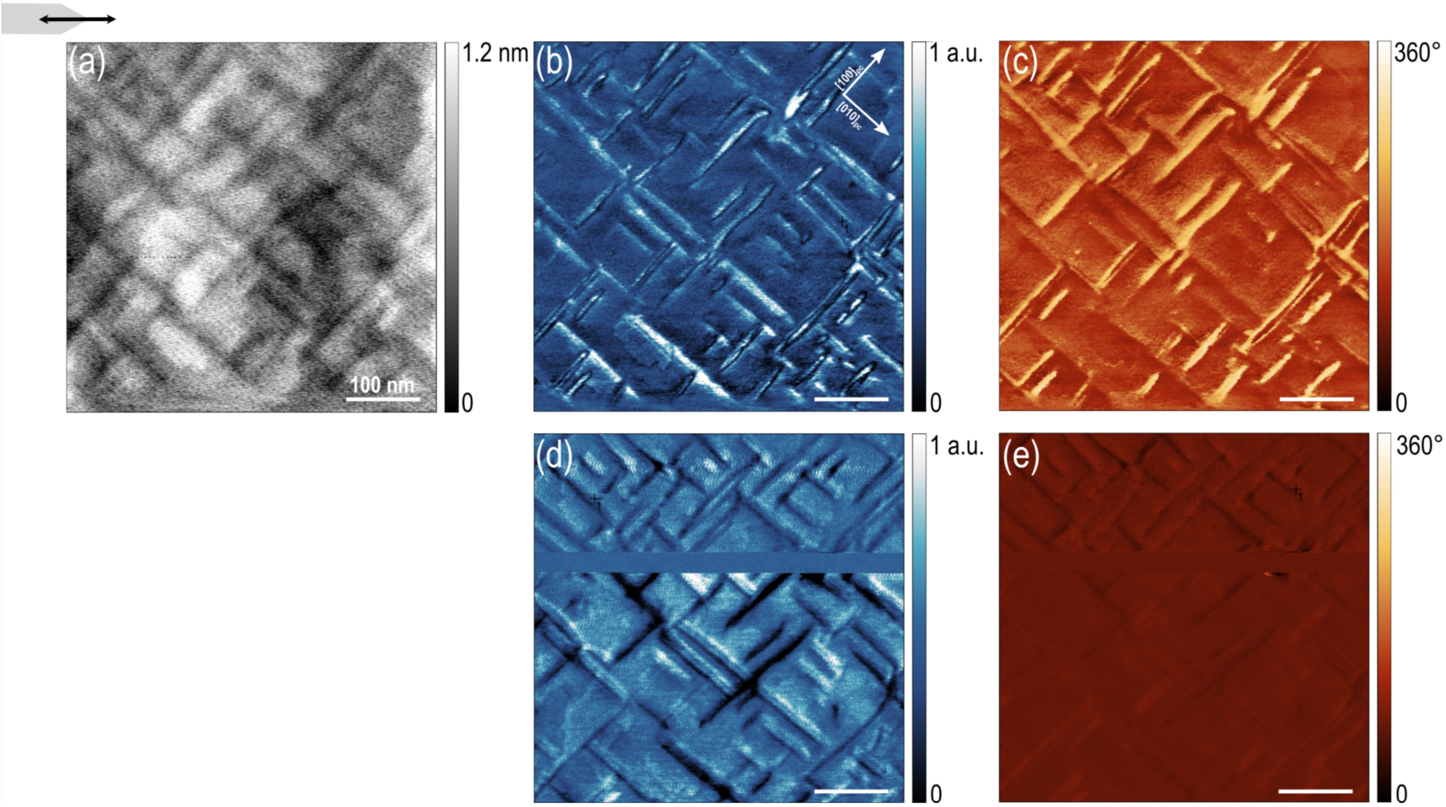

Figure S10: PFM analysis performed on the $PbTiO_3/SrRuO_3$ membrane in a flat region between ripples. (a) Topography image. (b,c) Lateral PFM amplitude and phase signals. (d,e) Vertical PFM amplitude and phase signals. These indicate the presence of an a/c domain structure.

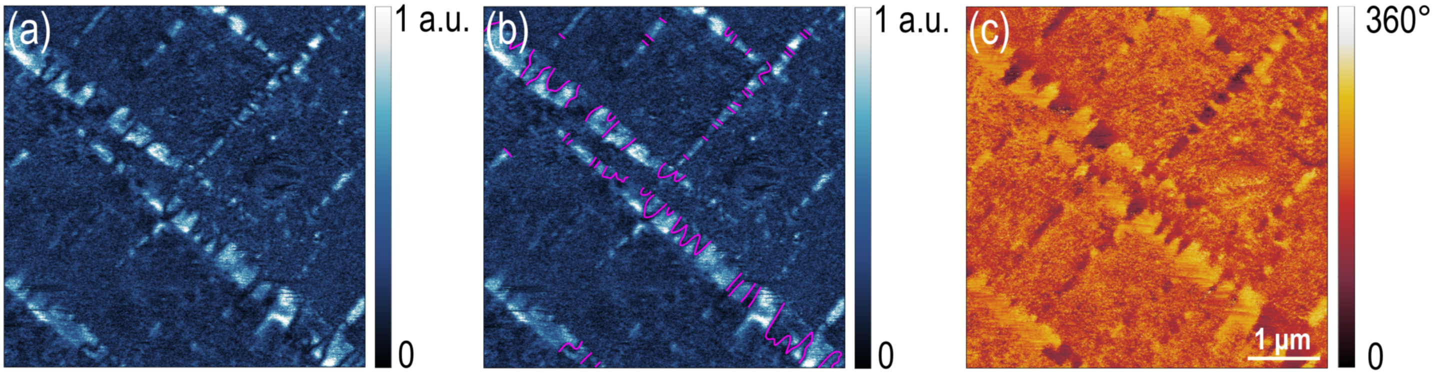

Figure S11: (a) Lateral PFM amplitude signal, as shown in Figures 3d of the main manuscript. (b) Schematic of the domain walls configuration at the ripples. The 180° domain walls, separating domain with opposite polarization orientations, are indicated in pink. (c) Lateral PFM phase signal, as shown in Figures 3e of the main manuscript.

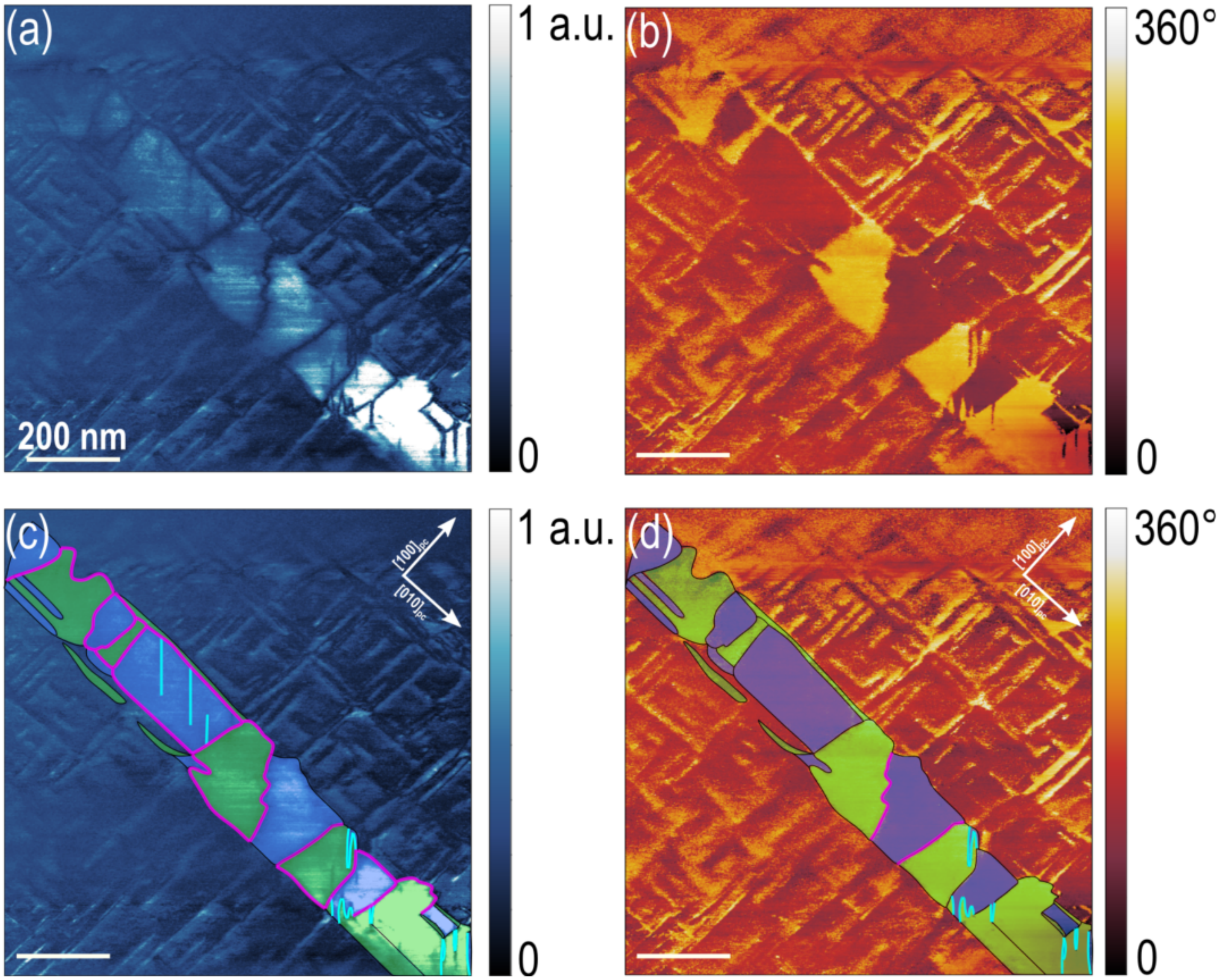

Figure S12: (a,b) Lateral PFM amplitude and phase signals, as shown in Figures 3f and 3g of the main manuscript. (c) Schematic of the domain configuration at the ripple. The 180° domain walls, separating a domains with opposite polarization orientations, and the 90° domain walls between a_1 and a_2 domains are highlighted by pink and blue lines, respectively.

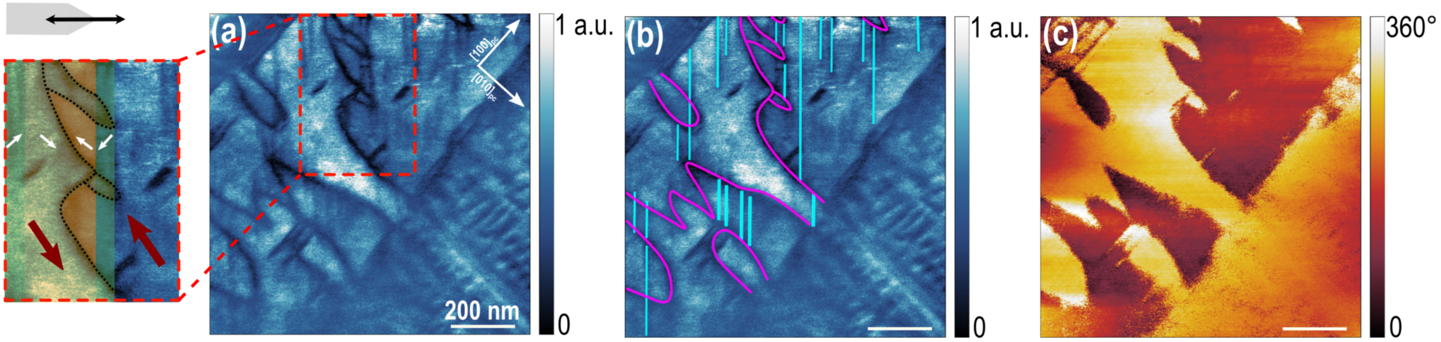

Figure S13: (a) Lateral PFM amplitude signal, as shown in Figures 3h of the main manuscript, showing the presence of a_1 and a_2 organized in superdomains. The sketch on the right shows the a_1 and a_2 domains within the superdomains, with their respective polarization directions (white arrows), the red arrows provide the direction of the resulting net polarization. (b) Schematic of the domain configuration at the ripple. The 180° domain walls, separating superdomains with opposite net polarization orientations, and the 90° domain walls between a_1 and a_2 domains are highlighted by pink and blue lines, respectively. (c) Lateral PFM phase signal, as shown in Figures 3i of the main manuscript, showing the superdomains with opposite net polarization orientation.

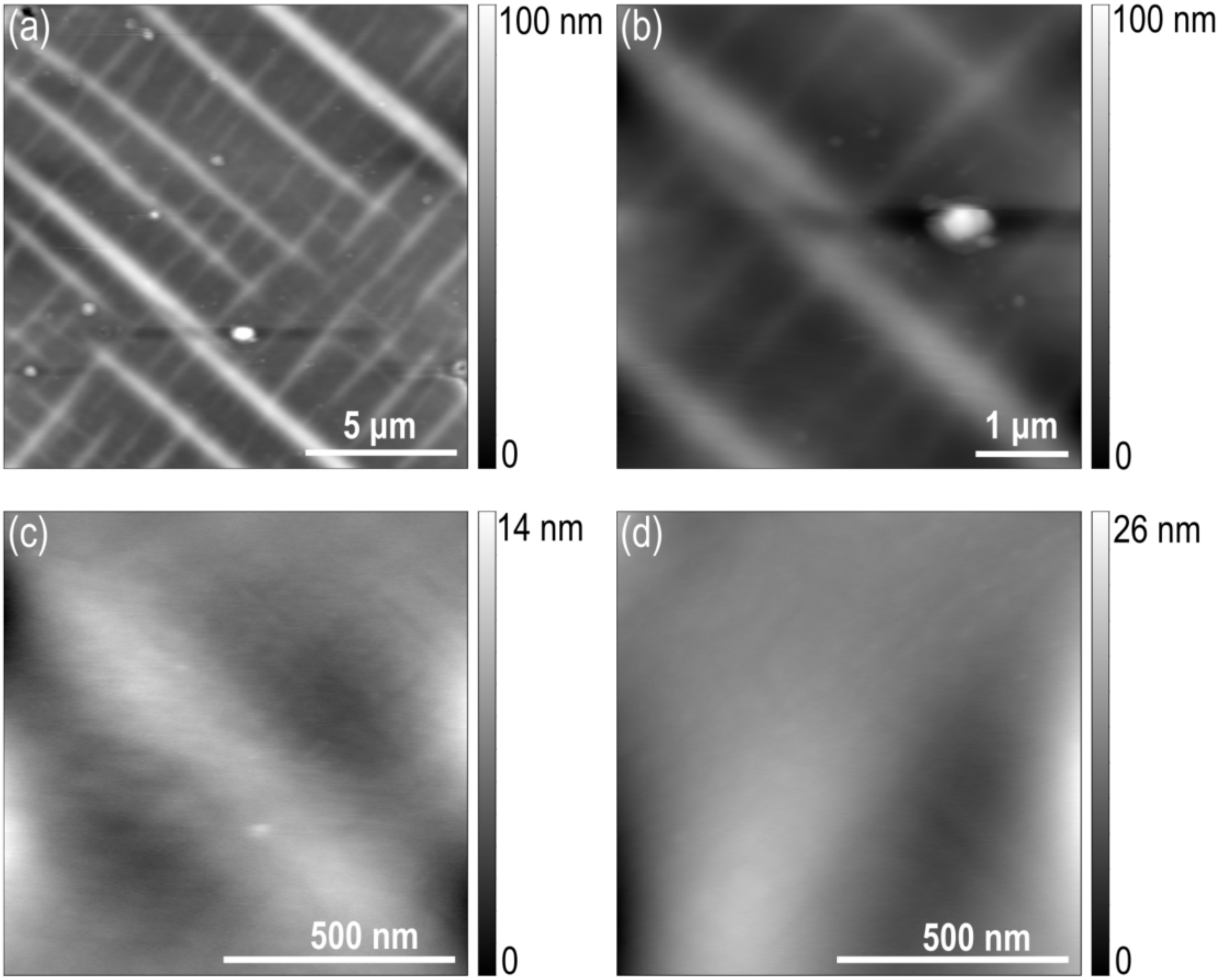

Figure S14: (a) Two-dimensional topography image of the 3D representation reported in Figure 3a of the main manuscript. (b) Topography image corresponding to the amplitude and phase signals reported in figure 3d and 3e of the main manuscript. (c) Topography image corresponding to the amplitude and phase signals reported in figure 3f and 3g of the main manuscript. (d) Topography image corresponding to the amplitude and phase signals reported in figure 3h and 3i of the main manuscript.

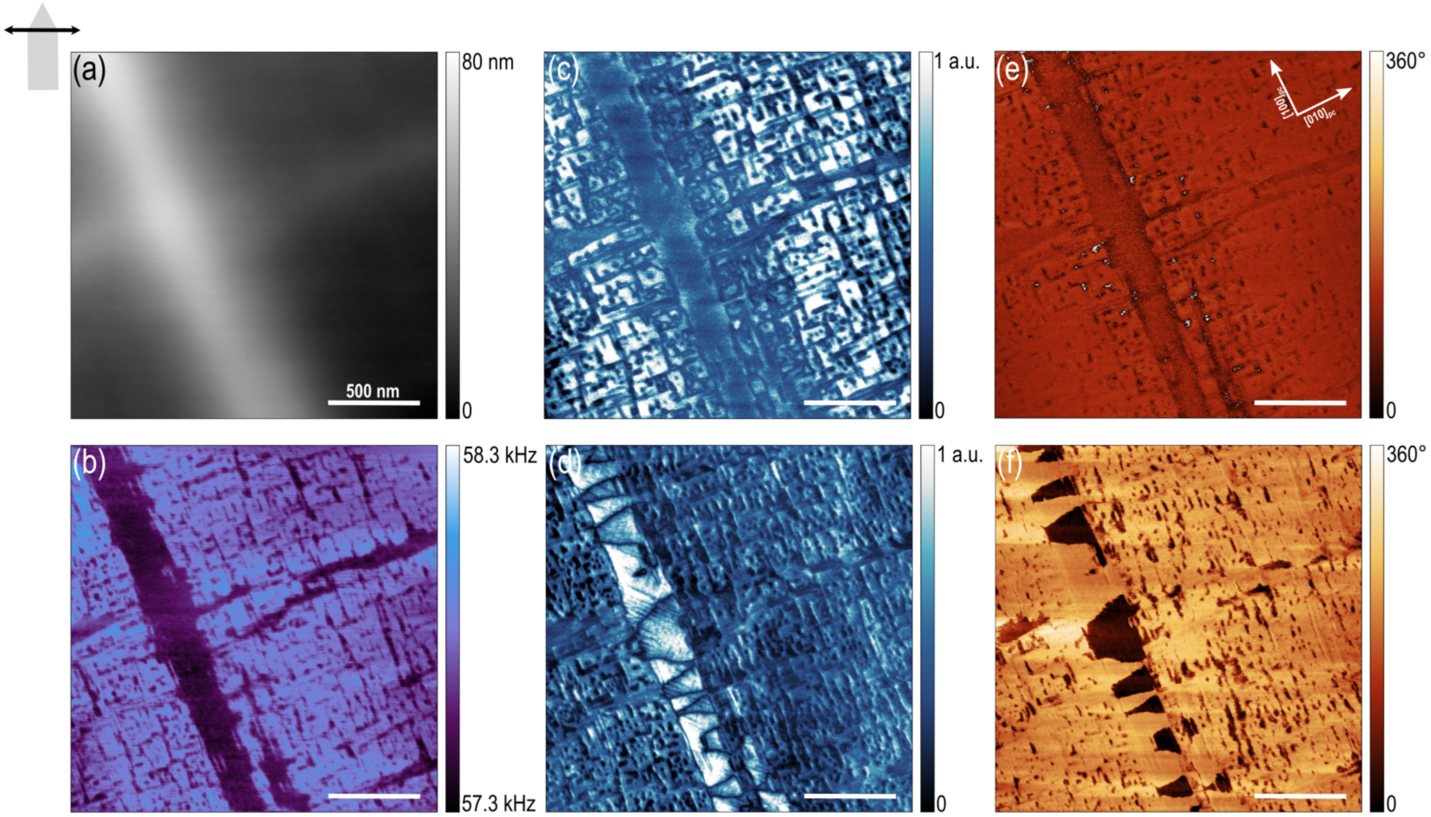

Figure S15: PFM and CRFM analysis conducted at the same location. (a) Topography image. (b) CRFM signal presented in Figure 6a of the main manuscript. (c,d) Vertical and lateral PFM amplitude signals. (e,f) Vertical and lateral PFM phase signals.

References

[1] Mark Fox. *Optical properties of solids*, volume 3. Oxford university press, 2010.

[2] Robert W Boyd, Alexander L Gaeta, and Enno Giese. *Nonlinear optics*. Springer, 2008.

[3] Chang Jae Roh, Sun Young Hamh, Chang-Soo Woo, Kwang-Eun Kim, Chan-Ho Yang, and Jong Seok Lee. Ferroelectric domain states of a tetragonal bifeo$_3$ thin film investigated by second harmonic generation microscopy. *Nanoscale Research Letters*, 12:1–6, 2017.

[4] Chang Jae Roh, Jin Hong Lee, Kwang-Eun Kim, Chan-Ho Yang, and Jong Seok Lee. Deterministic domain reorientations in the bifeo$_3$ thin film upon the thermal phase transitions. *Applied Physics Letters*, 113(5), 2018.

[5] Pauli Virtanen, Ralf Gommers, Travis E. Oliphant, Matt Haberland, Tyler Reddy, David Cournapeau, Evgeni Burovski, Pearu Peterson, Warren Weckesser, Jonathan Bright, Stéfan J. van der Walt, Matthew Brett, Joshua Wilson, K. Jarrod Millman, Nikolay Mayorov, Andrew R. J. Nelson, Eric Jones, Robert Kern, Eric Larson, C J Carey, İlhan Polat, Yu Feng, Eric W. Moore, Jake VanderPlas, Denis Laxalde, Josef Perktold, Robert Cimrman, Ian Henriksen, E. A. Quintero, Charles R. Harris, Anne M. Archibald, Antônio H. Ribeiro, Fabian Pedregosa, Paul van Mulbregt, and SciPy 1.0 Contributors. SciPy 1.0: Fundamental Algorithms for Scientific Computing in Python. *Nature Methods*, 17:261–272, 2020.
